# Psychological characteristics of students in learning clinical interview skills with the use of virtual patient

**DOI:** 10.1186/s12909-020-02344-6

**Published:** 2020-11-17

**Authors:** Bartosz Zalewski, Maciej Walkiewicz, Mateusz Guziak

**Affiliations:** 1 Department of Psychology of Individual Differences, Diagnosis and Psychometric Research, SWPS University of Social Sciences and Humanities, Warsaw, Poland; 2 Department of Psychology, Medical University of Gdańsk, Tuwima 15 Street, 80-210 Gdańsk, Poland; 3 Faculty of Medicine, Medical University of Gdańsk, Gdańsk, Poland

**Keywords:** Interview, Decision-making, Clinical reasoning, Virtual patient, Simulation, Assessment of clinical skills

## Abstract

**Background:**

The goal of this exploratory study is to analyse which psychological characteristics of students are related to the effectiveness of learning clinical interview skills with the use of a virtual patient (VP).

**Methods:**

The sample consisted of 29 final-year clinical psychology students. The authors’ VP tool was used for measuring and teaching clinical interview skills: building contact with the patient, gathering important information, and identifying the students’ mistakes. Psychological questionnaires were used to measure the students’ psychological features: need for cognitive closure, ability to achieve cognitive structure, beliefs in the changeability of human traits, level of hope, intelligence, positive vs negative affect, and academic knowledge.

**Results:**

The most important aspect of the diagnostician’s psychological features which substantially influence effectiveness of learning interview skills is belief in the stability or changeability of human traits and the need to achieve cognitive closure. Participants who have a belief in human changeability are able to perform the task correctly even without training, while those who believe in human stability improve only slightly with training. Students with lower need of cognitive closure successfully learned to build a good relation with the patient.

**Conclusions:**

The study allows a better understanding of the phenomena occurring during the learning of clinical interview skills with the use of a VP.

**Supplementary Information:**

The online version contains supplementary material available at 10.1186/s12909-020-02344-6.

## Background

The diagnostic process is a complex, patient-centred, collaborative activity that involves building contact with the patient and gathering diagnostic information [1, 2]. Building contact is the leading task, enabling the collection of relevant information that will be used to build diagnostic hypotheses [2]. The clinical interview concerns a set of questions describing the problem, such as: examples of a problem situation, how the subject understands his or her symptoms, expectations of treatment and resources [3]. An effective interview structure ensures that all areas of potential clinical concerns are assessed [4]. What is more, it is of high importance to avoid mistakes, e.g.: giving interpretations or advices instead of questions, offering suggestions, providing evaluations of patient behaviours [1].

The literature shows little interest in the matter of the individual psychological characteristics such as basic cognitive and affective processes in the context of clinical interview [5–9]. Nonetheless, it significantly influences the processes of learning and realisation of cognitively complex and emotionally burdening tasks such as clinical interview [10].

Available data indicate significant psychological features that might help in developing interview skills. The need for cognitive closure (NFC) is defined as the desire for an answer on a given topic, compared to confusion and ambiguity [11, 12]. Individuals with a high NFC have more focus and selective attention spans compared to their low-NFC counterparts [13]. In the context of decision making, a high NFC is related to a more limited information search before making a decision [14], higher ratings of confidence after making a decision [15] and a stronger preference for familiar choices instead of new options [16] compared with a low NFC [17]. High levels of NFC are associated with deficits in the area of working memory [18], low tolerance of ambiguity and a tendency to stereotype and to have prejudice [19]. What is more, in general, processes important for the development of interview skills are: less extensive search for information, limited number of generated hypotheses, primacy effect in impression formation, numerical anchoring, or preference for quick decision making, usually interpreted as a general reluctance to invest effort in judgements and decision making [11].

Another construct which can impact on the development of interview skills is the ability to achieve closure (AAC) which refers to the individual’s ability to reach swift decisions and structure in life. The level of the AAC can be understood as the extent to which individuals are able to use different styles of information processing according to their NFC [20]. High AAC individuals are capable of fulfilling their NFC and exhibit information processing that is consistent with their needs. Low AAC individuals with a high NFC are more motivated to use stereotypical thinking; they will gather inconsistent information or form one-sided and unambiguous categorisations [21]. These individuals may feel overwhelmed with excessive choice in everyday decision making and experience it as stressful. Low AAC and high NFC is associated with impaired mental health [22].

Beliefs in changeability vs stability of human traits [23] could also influence the way students learn interview skills. This trait is meaningful for the level of motivation, tendency to take up challenges, social-emotional functioning and engagement in effortful tasks, which translates into, for instance, educational achievements and the ability to cope with stressful situations [24]. Individuals who believe in the changeability of human traits react in a more positive way to feedback concerning a change in the level of task performance [25]. In this context, beliefs of changeability vs stability seem to influence effective learning of diagnostic competence.

A high level of hope fosters psychological wellness, which involves taking up challenges and achieving goals that are important for the individual. A high level of hope can thus be related to a readiness to invest more resources in the task, which can prevent the negative influence of anxiety [26].

Positive affect/negative affect*.* Positive affect is associated with better cognitive functioning and can improve verbal fluency performance [27, 28], a higher performance level of tasks requiring motor skills, auditory-visual coordination and abstract thinking [29]. It has also been shown that positive affect is related to a higher level of cognitive control [30], as well as a higher level of problem solving and decision making abilities, facilitating cognitive processing which is flexible, creative and effective [31]. However, the findings from research on the positive influence of affect on cognitive functioning are not univocal, it is, for instance, known to decrease the ability of cognitive inhibition in relation to an emotionally marked material [32].

Level of academic knowledge. In the situation of realising a cognitively complex task which involves analysing a lot of data of diverse significance, the limited abilities of conscious information processing constitute an important difficulty [33]. What is helpful in dealing with the limited abilities of conscious data processing is the knowledge possessed – in a situation requiring analysing complex problems, activating the knowledge gathered in the long-term memory enables a resource-saving structuring of the incoming data [34]. Conducting a clinical interview is a very complex cognitive task [35]. Therefore, it seems necessary to control for the intelligence level of the participants.

It is important to mention that one of the most promising methods in the area of teaching and evaluating clinical interview skills is the virtual patient (VP). It is defined as Interactive Patient Scenarios for health, treatment, education and measurement purposes [36]. VP technology has become one of the most common types of problem-based learning in medical education [37]. This concept incorporates interactive computer simulators that are used in medical education, and their main focus is on simulating the clinical steps including: patient history, physical examination, laboratory tests, diagnosis, treatment prescription, and feedback study of virtual patient applications [38]. What is more, these programs also include communication skills which form the basis of clinical practice [39]. Integrated lower cognitive-emotional burden is characteristic for this type of training, since the student performs the diagnostic tasks without a direct contact with the patient. VP helps students to integrate theoretical knowledge and clinical experience, providing structure that prepares students for the open-structure clinical environment and patient encounter [40]. Despite current technological limitations, the VP can provide students with a controlled and secure learning environment with the possibility of continuous and modifiable practice with feedback without negative consequences for real patients [41].

### Goal of the study

The goal of this exploratory study is to analyse which psychological characteristics of clinical psychology students are related to the effectiveness of learning clinical interview skills with the use of a virtual patient (VP).

## Methods

### Participants

The sample consisted of 29 final-year clinical psychology students at the SWPS University of Social Sciences and Humanities, Warsaw, Poland (average age M = 28.48; Me = 24; SD = 8.53; 86% female). Participation in the study was one of the options for completing an obligatory student internship. Students completed some of the hours required for the internship, however, they did not receive any payment. Respondents could withdraw from participation at any time. We noted that 24% of participants dropped out from the research due to multi-stacking, length and complexity of the study.

### Procedure

**Fig. 1:**
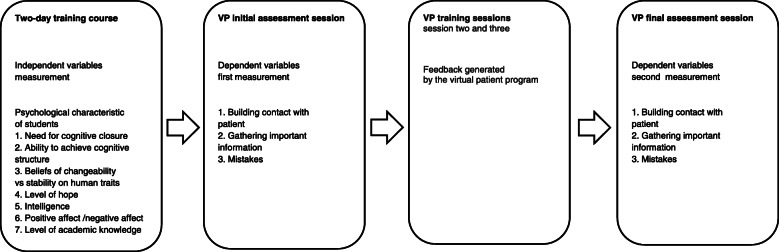
The study procedure

The study was performed between September 2017 and May 2018. The participants took part in a two-day training course (12 h) of clinical interview and decision-making skills. The psychological features of students were measured (independent variables). In the next step, there were four VP sessions, each consisted of completing a VP clinical interview. The goal of the initial session was to measure the primary level of students’ clinical interview skills (dependent variables) using the VP program as an assessment tool. The second and third sessions were training sessions when the VP was used as a teaching tool. During the final session, the VP program was used as an assessment tool for measuring the final level of clinical interview skills (Fig. 1).

### Tools

The *independent variables* (psychological features affecting the learning process) were measured by a series of questionnaires and tests:

1. The Need for Closure (NFC) Scale measure the cognitive-motivational aspects of decision making. NFC is defined as the desire for an answer on a given topic, compared to confusion and ambiguity. Respondents rated 27 items on a six-point scale (from 1 = completely disagree, to 6 = completely agree). The mean score of all items was calculated (Cronbach α =0.84). The higher the mean score, the higher the need for closure (NFC) [15].
2. Efficacy at Fulfilling the Need for Closure (EFNC) refers to the individual’s ability to reach swift decisions and structure in life. Scale achieves good psychometric properties [20].
3. Implicit Self-Theory Scale. The 8-item scale has also demonstrated good construct validity with scores predicting theoretically meaningful relationships with a range of variables [42].
4. Basic Hope Inventory (BHI-12) consists of 12 statements: the examinee defines to what extent s/he agrees with each of them – for that purpose s/he uses a 1 to 5 scale (where 1 = “I definitely disagree” and 5 = “I definitely agree”). Reliability: satisfactory internal consistency and stability were found out in the examination of older high-school students. Validity: The BHI-12 scores are positively correlated with personality variables referring to good adjustment (e.g. optimism, life satisfaction, internal sense of control, openness to experiences) and negatively with anxiety and depressiveness. The relationship was also found out between basic hope as measured by the BHI-12 and constructive and pro-developmental responses to difficult situations. Factor analysis of the questionnaire revealed (in accordance with theoretical assumptions) two factors of which the first refers to beliefs of the world’s friendliness and the second to those of the world’s order and predictability [43].
5. Intelligence – Raven Progressive Matrices being a non-verbal intelligence test consists of 60 items divided into five Series (A, B, C, D, E), each comprising 12 items. Each item consists of an incomplete pattern (matrix) and the subject is requested to find the missing bit out of the six pieces shown beneath the matrix. Reliability: high internal consistency and stability. Validity: significant correlations with school grades and WISC-R scores for children and adolescents [44, 45].
6. Positive and Negative Affect Schedule (PANAS) measures the strength of negative or positive emotions. Participant has to assess, using 5-point scale, to what extent the given adjectives corresponds with her/his current state or with usual emotional traits. Reliability: internal consistency of the scales is high or satisfactory – Cronbach’s alpha coefficients, depending on the version and the kind of sample, oscillate between .73 and .95. Validity: proven on the basis of factor analysis, cluster analysis, correlations with other tools, and intergroup differences [46, 47].
7. *Level of academic knowledge* – single-choice test (20 questions, 20 min) at the end of the two-day training course.

The *dependent variables* (the level of students’ clinical interview skills) were as follows:

1. *building contact with patient*,
2. *gathering important information,*
3. *mistakes.*

These variables were measured by the authors’ VP tool. The VP was designed for both assessment and teaching interview skills. The VP consisted of short recordings of a person playing the role of patient in a consultation for psychological treatment, constituting a 20–25 min diagnostic interview (videos + participant’s reactions). Students watched 9 successive fragments of the patient interview, and after each fragment, they chose one of two diagnostician interventions (better or worse). Depending on the decision, another fragment was launched. In total, the program enabled 512 combinations of selection paths, arranged in a decision tree. In the VP teaching sessions (sessions two and three), the students received feedback after each decision. It contained information about the appropriateness of the chosen intervention with an explanation and guidelines for further steps. The VP used a simple algorithm to count each selection of the participant assigning the appropriate number of points to his/her choice. Scoring was used to calculate the effectiveness of the interview (VP as assessment tool). The authors’ clinical material is based on the Keyes and Lopez dimension model [3, 48]. The VP's patient problems described in their model were based on a patient struggling with increasing problems that correspond to the level of personality disorders. We decided to use the VP presenting symptoms of such serious disorders because the pilot study revealed that it is difficult for students to recognise mental disorders of lower severity (Additional file 1).

### Statistics

IBM SPSS software was used for data analysis. Pearson correlations were conducted to investigate relationship among the data.

## Results

The *Need for cognitive closure* presents a moderate negative correlation with *building contact with patient* in the initial interview. The *ability to achieve cognitive structure* presents a moderate negative correlation with *building contact with patient* in the final session and a low negative correlation with *gathering important information* in the initial interview. The *beliefs of changeability of human traits* presents a moderate positive correlation with *gathering important information* in the initial and final sessions; and a moderate negative correlation with *mistakes* in the initial session. The *positive affect* presents a moderate negative correlation with *building contact with patient* in the final session (Table 1).

**Table 1:** Relations between psychological features of diagnosticians and clinical interview skills learned with the use of virtual patient

	Building contact with patient	Gathering important information	Mistakes
**1. Need for cognitive closure**	I - - - r(15) = −0.534; *p* = 0.041		
**2. Ability to achieve cognitive structure**	F - - - r(15) = −0.580; *p* = 0.023	I - - r(24) = −0.424; *p* = 0.039	
**3. Beliefs of changeability on human traits**		I + + + r(23) = 0.585; *p* = 0.003 F + + + r(15) = 0.552; *p* = 0.033	I - - - r(24) = −0.550; *p* = 0.005
**4. Level of hope**			
**5. Intelligence**			
**6. Positive affect/negative affect**	F - - - r(16) = −0.733; *p* = 0.001		
**7. Academic knowledge**			

I - initial session, F - final session

+ + + + .70 to .90 high positive correlation

+ + + .50 to .70 moderate positive correlation

+ + .30 to .50 low positive correlation

+ .00 to .30 negligible correlation

- - - - −.70 to −.90 high negative correlation.

- - - − .50 to −.70 moderate negative correlation.

- - − .30 to −.50 low negative correlation.

- .00 to −.30 negligible correlation.

## Discussion

In general, our study shows that psychological characteristics of students are related to the effectiveness of learning clinical interview skills, when using digital tools like VP. The most relevant inner student characteristics was the belief in stability vs changeability of human traits. In relation to the analysed clinical interview skills, belief in changeability enhances proper collection of diagnostic information, it is not associated with building interpersonal contact and is related with committing a low number of mistakes.

By definition belief in the changeability of human traits means a higher tolerance of complexity and contradictions, and a greater tolerance of one’s own mistakes [23]. At the same time, this feature favours the clinician’s curiosity, his/her desire to collect more information and to understand the patient as a complex person. Certainly, for such students it is natural to receive varied, often contradictory information from the patient, which cannot be quickly arranged into a clear diagnosis. People convinced of the stability of human traits tend to feel a higher level of anxiety in such a situation and try to reduce the complexity of the information to build an oversimplified picture of the patient [24]. Probably, they are also less tolerant of their mistakes which increases stress and the likelihood of making mistakes.

In our research diagnosticians convinced of the stability of human traits do not improve their effectiveness in obtaining information nor building contact with a patient but they reduce the number of mistakes. Students who believe in the stability of human traits tend to quickly get the feeling that they have identified and solved the patient’s problem. Their way of processing information is prone to a significant number of simplifications, often stereotypes. As a consequence, they do not need to collect further information and finally, they conduct less informative interviews. The university curriculum could support students convinced of the stability of human traits in obtaining more complex information during an interview.

The second most important characteristic is the ability to achieve cognitive structure. Students with a higher ability skipped gathering clinical information, but after training, they began to apply it. At the same time, they stopped building interpersonal contact. Feedback from the VP training may be helpful to understand these results as the program instructed them to collect more diagnostic information. As a result, they abandoned building contact. Perhaps, they devoted all their attention to collecting relevant information, but those with a lower need for cognitive closure successfully learned to build contact with the patient. Maybe the chaos and ambiguity accompanying the extraction of complex and unclear diagnostic information is not very burdensome for them [10]. During clinical interview training, we should particularly support students with a higher need for cognitive closure by offering them a well-functioning structure.

Another result is connected with positive affect which correlates with the level of contact with a patient. No impact on structure or making mistakes was noted. This is an interesting result, because positive affect usually increases cognitive flexibility and coping with complex tasks [31]. People with a high level of positive affect usually have weakened cognitive inhibition capabilities [32]. Perhaps, under the influence of the feedback obtained during VP training, students focus only on extracting information, so that they cannot slow down to enable the building of interpersonal contact with the patient. It is also interesting that no negative effects of experiencing a dominant negative affect were noted. It should theoretically ‘stiffen’ one’s thinking and impair one’s ability to perform the parallel tasks which are necessary in the clinical interview.

### Limitations

There were a small number of participants, which limits the possibility of generalising the results for a population of students. The study was conducted at one university and focused at clinical psychology students (in their final-year of study), thus limiting the transferability of the findings to general populations of clinicians. All the results and conclusions basically relate to the teaching of clinical interview skills with the use of virtual technology. Due to the multi-stage, length and complexity of the research, there was a high rate of dropouts.

## Conclusions

1. The study allows a better understanding of the phenomena occurring during the learning of psychological clinical interview skills with the use of VP.
2. The most important characteristic of the diagnostician was found to be a belief in the stability or changeability of human traits. Belief in changeability enhances the collecting of diagnostic information. Students convinced of the stability of human traits should be supported in obtaining more complex information during interview training.
3. Students with a higher need for cognitive closure may be supported by offering them a well-functioning structure. It is also worthwhile to provide highly structured learning tools to build contact with the patient.
4. Students with a high ability to achieve cognitive structure should focus on building contact with the patient.

## Supplementary Information

**Additional file 1.**

## Data Availability

The datasets used and/or analysed during the current study are available from the corresponding author on reasonable request.

## Abbreviation

VP: Virtual patient
