# Supplementary material for: Psychological characteristics of students in learning clinical interview skills with the use of virtual patient
Source: BMC Med Educ. 2020 Nov 17;20:441. doi: 10.1186/s12909-020-02344-6 (PMC7672874; doi:10.1186/s12909-020-02344-6)
Supplement: Supplementary file 1 — Additional file 1. [file 12909_2020_2344_MOESM1_ESM.docx]

**APPENDIX 1**

**Profile 1, Miss X, 33**

She comes because she would like to take better care of herself. She works as a choreographer. She is highly valued for her competences, but still gets the position of assistant, not the main production choreographer. She dreams of being noticed, discovered, but thinks that in this environment there are many jealous, unkind women who compete in a fair way. The patient would not like to compete the way they do - she values good relations, does not want to stand out at the expense of others. At the same time, she has a lot of very positive phrases about her professional competence. She would like to be able to effectively strive for a position that would reflect her competences, at the same time she would like to be able to do it her own way - without entering into an unhealthy competition.

Currently, she is in a relationship with her partner - D., who is a businessman and earns a lot. They live in a house near Warsaw. It is very beautiful there - a garden, a fountain, their own conservatory. However, being there, away from people, the city, numerous friends of the patient - is tedious. The patient works hard there conceptually - she declares that she works best among people. She organizes social life for herself and her partner. She describes his partner as cool, slightly withdrawn, showing feelings in a very reserved way. The patient is emotionally in a very difficult situation - tired of the relationship with a cool partner, for the first time in her life she got involved in an affair with her colleague M.. He is brilliant - very talented, sociable, affectionate. The patient feels that it is her "soul mate" - a man with a passion for life, with interests that are close to her - is like a male version of herself. The patient feels that she has been waiting for someone like that all her life. At the same time, she doesn't feel strong enough to end her relationship with her partner. She told him about the affair - she wants to be honest in her relationship, she has the rules. D. was angry, but he decided to forgive her and strive for their relationship. He says that the patient is the most important person in his life. The patient is trapped - she loves M. but doesn't want to be disloyal to D. Mom advises her to end the affair, commenting that the relationship with D. is yet another relationship in the patient’s life and it is not known if it will not be in the relationship with M. that after 2 or 3 years this relationship will get boring to the patient, and yet Miss X has already 33 years and it would be good to stay in a stable relationship.

The patient acknowledges her mother partly right, but she would not like to duplicate her story - my mother is in a very cold relationship with her father. The father is highly placed in the structures of a large state-owned company - he earns a lot, he is often not at home. He always emphasized that Miss X could not bring him shame. Miss X feels very sorry for her mother about this relationship - she understands that her mother had to look for warmth in relationships outside of marriage. However, she would like to be in a relationship with someone whom she loves and who shares her passions and energy for life and knows how to show her attachment.

Example of a situation (work)

Recently, the patient was employed in the production of a short film. She was very pleased with the results of her work, the director spared her praise. The production budget was large - it was visible, nothing was saved. The patient was employed by the head of choreography. After implementation, the boss delayed the transfer of remuneration for a long time - it was really frustrating. The patient later learned that she earned a fraction of what the boss did, although she did almost nothing - all the best ideas were Miss X's ideas. It arouses anger, indignation and powerlessness - the patient would not dare to apply for an independent position, contrary to the boss who had previously hired her.

Example of a situation (relations)

The patient came to Warsaw last week because of the promotion of the film with which she collaborated. D. forbade her to participate in the informal celebration that took place after the official event. Her friends were very surprised that she was gone, they started calling her. In the end, she gave in and joined them. M. was also there. They went for a walk together, talked for a long time - it was amazing as if they had known each other for many years. Later, however, the patient decided that she was behaving badly, she barely said goodbye and went to the friend she was supposed to sleep with, he was surprised that she came without M.. He suggested that they go home to M. together. It turned out that he was talking to M. on the phone, M. was inviting for wine and mussels. The patient decided that she could go. They arrived and a friend said goodbye after several minutes. The client finally spent the night with M. - it was incredibly close and significant, but she felt unfair towards D.

Protodiagnosis - "my father taught me certain principles, it is difficult for me when I cannot achieve my goals in accordance with the principle of being fair to others."

Expectations - get a job as the first choreographer. Be able to take care of her needs in her personal life - be good to choose in a way that does not hurt others.

**Profile 2, Miss Y, 26**

She is in a successful relationship, she would like to be able to start sex fully. Also, she is very tired in her work - she works in a clothing store in a large shopping center. She is highly valued at work, but she works over 12 hours a day, she doesn’t have free weekends, she often works at unfavorable hours, the boss is mobbing her, she is afraid of getting angry with the boss and does not refuse her. She is afraid that he will not find a better job. She is not strong enough to send a CV to another place. she studies journalism - she is doing great, she has a scholarship and a lot of support from the faculty authorities (lecturers appreciate her commitment and competence). Has a large group of friends and two close one. Together they go on vacation to Masuria. They like to read, talk and spend time together. Now she lives with her boyfriend. Arranging the common room gave them a lot of joy. They live with the patients’ boyfriend's father, who often goes away, does not interfere in their affairs - she and her partner often have the whole apartment for themselves. Recently they adopted a cat, they called him Harold. The client values ​​contact with the animal very much - she likes how Harold mutters and sits on her lap. The client is friends with her boyfriend's sister and her boyfriend.

Currently, she is afraid that her lack of intercourse will harm their relationship. She is afraid that her boyfriend will leave her, although he declares that this is not true. However, she thinks he can't stand it any longer. The boy finances her psychologist consultation and wants to finance her therapy because she earns little. The patient would like to be able to dare to change jobs. She would like to earn more so as not to be a burden to her boyfriend - she wants him to be able to afford nice clothes and enjoy himself.

When she thinks about changing jobs, she is afraid that people reading her CV will laugh at her lack of competence. She is afraid that even if she finds another job, she will not be better in it than in her current job, that she will be a victim of mobbing again. She feels - an irrational fear of men who remind her of a man who has molested her in the past. It was a father's friend, the patient does not want to talk about this anymore. She is afraid that she might meet someone similar to this man at an interview or a new job. She knows that it would paralyze her - she wouldn't speak, she couldn't work. She would like to undergo therapy focused on dealing with the fear of intercourse and the fear of looking for a new job. She emphasizes that she doesn't want to work with a sexologist because she thinks her problems are emotional, not physical. The patient had a relationship in which the boyfriend proposed to her. They lived together for several months. She did not have sex with him. It turned out that he was cheating on her.

The patient is afraid that a relationship without an element of intercourse is so unsatisfactory for a man that she expects that if she soon won't feel able to have sex with her boyfriend, he will start cheating on her or will leave her.

An example of a situation in which the patient was afraid of leaving her boyfriend

Recently, the boy came to work for her. He noticed a dress in her shop and asked her to try it on. The patient put on a dress and noticed that she looks attractive in it. The boy said that he looks great. However, she thought that she was now attractive to the boy and she felt horror - a tightness in the stomach and tension in the arms and neck. She thought that he would like to have sex with her, and she is very afraid of it, she does not want it very much. She thought about her reaction as abnormal - women like to please their partners. She felt guilty, strange. She thought her boyfriend wouldn't want to tolerate it in the long run. She told him that she did not like this dress and immediately hung it up. The whole evening was tense and sad, she wanted to cry. At the same time, she tried to hide it from her boyfriend.

An example of a situation in which the patient was afraid to look for a new job

Yesterday the patient looked at job offers in the newspaper. She began to check if she had the competencies required for several positions that seemed attractive to her. First, she decided that she actually had these competencies, and then she imagined that she would be asked about them during the interview (e.g. the interview would be in English). She imagined how she was losing the thread, and the interviewer comments on her lack of skills and accuses her of lying - she wrote that she knew English. The patient feels fear paralyzing her - she pushes the newspaper away and decides not to think about it anymore.
